# Supplementary material for: Ethical, Legal, Organisational and Social Issues of Teleneurology: A Scoping Review
Source: Int J Environ Res Public Health. 2023 Feb 19;20(4):3694. doi: 10.3390/ijerph20043694 (PMC9962592; doi:10.3390/ijerph20043694)
Supplement: Supplementary file 1 [file ijerph-20-03694-s001.zip › Supplementary File S1.pdf]

# Supplementary File S1: Participants attending the online meeting

**Patient associations**

| Name and surname | Organization |
| --- | --- |
| Nieves Rodríguez | Federación Española de enfermedades neuromusculares (ASEM) |
| Mónica Rodríguez Rubio de la Torre | Fundación ONCE |
| Laura Carrasco Marín | Federación madrileña de enfermedades neurológicas (FEMADEN) y Asociación Parkinson Madrid |
| Alfonso Castresana | Esclerosis Múltiple España (EME) |

**Scientific societies**

| Name and surname | Organization |
| --- | --- |
| Joan Ferri Campos | Sociedad española de neurorrehabilitación (SENR) |
| Gregorio Gómez Soriano Juan Carlos Oliva Pérez | Sociedad Española de Informática de la Salud (SEIS) |
| José Serratosa | Sociedad Española de Epilepsia |

**Clinical experts**

| Name and surname | Organization |
| --- | --- |
| María Esther Cubo Delgado | Servicio de Neurología, Hospital Universitario de Burgos. Burgos. |
| Raixa Noemí Pérez Martín | Servicio de Innovación Organizativa y Transformación del Modelo Asistencial.<br>Gerencia Regional de Salud. Junta de Castilla y León. Valladolid. |

**Industry**

| Name and surname | Organization |
| --- | --- |
| María Álvarez Orozco Valeska Seguel | Medtronic Ibérica, S.A. |
| Miren Bagüés | Tunstall Televida |

| | |
|---|---|
| Concha Toribio | Fenin |
| Francesc Mateu | Phenium |
