# Supplementary material for: Ethical, Legal, Organisational and Social Issues of Teleneurology: A Scoping Review
Source: Int J Environ Res Public Health. 2023 Feb 19;20(4):3694. doi: 10.3390/ijerph20043694 (PMC9962592; doi:10.3390/ijerph20043694)
Supplement: Supplementary file 1 [file ijerph-20-03694-s001.zip › Supplementary File S2.pdf]

Supplementary File S2: The semi-structured script of the online meeting with stakeholders

1. Introductions and purpose of the meeting.
2. Health technology assessment (HTA) in the National Health System
3. HTA Report on Teleneurology.
4. Discussion on the identification of problems or benefits on teleneurology and formulation of research questions to be included in the report.

- *What are the risks and benefits that, from your perspective, the use of teleneurology may have as a complementary service in the specialized care received by people suffering from a neurological condition?*
- *For which people is it desirable to introduce teleneurology? For which not?*
- *For what specific problems can teleneurology be appropriate? For which ones it is not?*
