# Supplementary material for: Ethical, Legal, Organisational and Social Issues of Teleneurology: A Scoping Review
Source: Int J Environ Res Public Health. 2023 Feb 19;20(4):3694. doi: 10.3390/ijerph20043694 (PMC9962592; doi:10.3390/ijerph20043694)
Supplement: Supplementary file 1 [file ijerph-20-03694-s001.zip › Supplementary File S3.pdf]

Supplementary Files S3: Search strategy for ethical, legal, organizational, social and patient, and environmental aspects

MEDLINE

1 Telemedicine/
2 Remote Consultation/ or computer communication networks/ or remote sensing technology/ or exp telephone/ or electronic mail/
3 (teleconsult* or telecare or telemed* or telerehab* or telehealth* or tele-medicine or tele medicine or telehealth or "tele-health" or telehomecare or "tele-homecare").ti,ab.
4 (mhealth or ehealth or "e-health" or "m-health" or m health or mobile health).ti,ab.
5 Videoconferencing/
6 (videoconferencing or conferencing or videoconference$ or "video-conferenc$" or videoconsultation or "video-consultation").ti,ab.
7 Mobile Applications/
8 ((mobile or cell$ or smart$) adj (app$ or device or phone)).ti,ab.
9 1 or 2 or 3 or 4 or 5 or 6 or 7 or 8
10 Neurology/
11 Nervous System Diseases/
12 (neurological adj (disorder* or disease*)).ti,ab.
13 stroke.ti,ab.
14 epilep*.ti,ab.
15 parkinson*.ti,ab.
16 multiple sclerosis*.ti,ab.
17 (nervous system adj (disorder* or disease*)).ti,ab.
18 movement disorder*.ti,ab.
19 Stroke/
20 Epilepsy/
21 Parkinson Disease/
22 Multiple Sclerosis/

23 Movement Disorders/

24 10 or 11 or 12 or 13 or 14 or 15 or 16 or 17 or 18 or 19 or20 or 21 or 22 or 23

25 9 and 24

26 telestroke*.ti,ab.

27 teleneurol*.ti,ab.

28 ((telemedicine or telehealth*) adj3 neurolog*).ti,ab.

29 25 or 26 or 27 or 28

30 ((("semi-structured" or semistructured or unstructured or informal or "in-depth" or indepth or "face-to-face" or structured or guide) adj3 (interview* or discussion* or questionnaire*)) or (focus group* or qualitative or ethnograph* or fieldwork or "field work" or "key informant")).ti,ab. or interviews as topic/ or focus groups/ or narration/ or qualitative research/

31 exp Ethics/ or ethics.fs. or (ethic* or bioethic* or unintended consequences or (skills and training) or (benefits and harms) or beliefs or patient* autonomy or dignity or moral* or religio* or human rights or humanism or patient* integrity or principlism or normativ* or principle-base* or beneficence or non-maleficence or philosoph* or aristoteles or socrates or justice or fairness or patient* expectation or patient* accessibility or Beauchamp or childress or wide reflective* or socratic or social shaping or casuistry or coherence analy* or eclectic* or right to die or right to life or social value* or ethnic value* or personal value* or benefit-harm or harm-benefit or elsi or elsa).ab. /freq=2

32 exp Privacy/ or exp Disclosure/ or exp Fraud/ or exp Government Regulation/ or exp Healthcare Disparities/ or exp Health Services Accessibility/ or exp Humanism/ or exp Human Rights/ or exp Insurance Coverage/ or exp Insurance, Health, Reimbursement/ or exp Jurisprudence/ or exp Legislation as Topic/ or Legislation & Jurisprudence.fs. or exp Patient Rights/ or exp Personhood/ or exp Prejudice/ or exp Professional Autonomy/ or exp Professional Misconduct/ or exp Social Stigma/ or exp Social Values/ or exp Stereotyping/ or exp Uncertainty/ or (((Healthcare or Health Care or nonclinical or Community Based) adj (Deliver* or Distribution* or System*)) or (legal* or liabilit* or litigation* or constitutional or justice or law or laws or jurisprudence or complicit* or privacy or private or confidential*) or ((care or treatment) adj2 (duty or obligat*)) or

(social* adj (responsibl* or obligat*)) or ((informed or presumed) adj2 (choice or decision making))).ti,ab,kf. or (basic right* or access* right or duty to know or equally access* or external pressure or fundamental right* or human right* or informed choice or informed decision making or public pressure or regulatory frame* or right of access* or right to know or acceptance or accessibility or accountability or autonomy or beneficence or civil right* or communitarian* or community values or confidentiality or coverage or dignity or directive or disclosure or discrimination or elsi or elsis or equality or equity orfairness or freedom or harm or inequalit* or inequity or patient* integrity or justice or law or lawmaker* or lawsuit* or lawyer* or legacy or legal* or legislation or legitimacy or liability or litigation or medicolegal or non-coverage or nonmaleficence or non- maleficence or normative or normativity or permissibility or prejudice* or privacy or reimburse* or social values or stigma or stigmas or stigmatisation or stigmatise or stigmatization or stigmatize or transparency or uncertainty or value judgment).ab. /freq=2

33 Decision Making, Organizational/ or Efficiency, Organizational/ or Models, Organizational/ or Organizational Objectives/ or exp Personnel Management/ or exp Professional Practice/ or "Organization and Administration"/ or Healthcare Common Procedure Coding System/ or Case Managers/ or Program Development/ or "Organization and Administration".fs. or organizational.ti,ab,kw. or ((organization* or work or workflowor work flow or work planning or implementation* or informationneeds or need assessment* or skills or attitud* or culture or patient path or user path or client path or quality assurance or sustainability or centralization or decentralization or hospital management or manager* or supplier*) adj9 (patient* or client* oruser or users or practice* or hospital* or home* or primary care or clinical or Medical or nurse* or physician* or profesional*)).ti,ab.

34 ((social or burden* or Impact* or choice* or information needs or communication* or self-care or self-management or trade-off) adj5 (social or patient*)).ti.

35 exp patient acceptance of health care/ or caregivers/ or exp Patient Preference/ or exp Patient Satisfaction/ or ((Patient* or individual* or person* or care-giver* or caregiver* or client or

communit* or consumer* or public* or user* or sufferer* or suffering or diseased or troubled or sick* or invalid or people or patient* or individual* or person* or carer or caregiver* or client or communit* or consumer* or public* or user* or sufferer* or suffering or diseased or troubled or sick* or people or patient or patients or proband* or individuals or survivor* or family or families or familial or kindred* or relative or relatives or care giver* or caregiver* or carer or carers or personal or spous* or partner or partners or couples or users or participant* or people or child* or teenager* or adolescent* or youth or girls or boys or adults or elderly or females or males or women* or men or men's or mother* or father* or parents or parent or parental or maternal or paternal) adj5 (prescrib* or Elicit* or Choice* or Logit or Adheren* or Preferen* or service-user or preference* or opinion* or perception* or perspective* or view* or voice* or experienc* or expectation* or wish or wishes or attitud* or lifespan* or refus* or patient autonomy or activities of daily living or quality of life or everyday life)).ti,kw.

36 ((patient* or individual* or person* or carer or caregiver* or client or communit* or consumer* or public* or user* or sufferer* or suffering or diseased or troubled or sick* or people or patient or patients or proband* or individuals or survivor* or family or families or familial or kindred* or relative or relatives or care giver* or caregiver* or carer or carers or personal or spous* or partner or partners or couples or users or participant* or people or child* or teenager* or adolescent* or youth or girls or boys or adults or elderly or females or males or women* or men or men's or mother* or father* or parents or parent or parental or maternal or paternal) adj2 (service-user or preference* or opinion or perception* or perspective* or view* or voice* or experience* or expectation* or wish or wishes or attitud* or Wellbeing or well-being or self-care or self* or belief* or concern* or worr* or burden* or problem* or distress or psychology* or social activit* or famil* or friend* or emotion* or satisf* or dissatisf* or happ* or unhapp* or behav* or lifestyle or routine or life or autonomy or activities of daily living or quality of life or everyday life or skeptic* or enthusias* or daily lives or frequent-or-daily-users or Acceptanc*)).ti,kw.

37 35 or 36

38 exp *pollution/ or exp *pollutant/ or *environmental exposure/ or exp *environmental impact/ or *ecotoxicology/ or *exp biota/ or exp *environmental health/

39 (waste* or pollution* or polluting or pollutant* or contamination* or contaminated or environmental health).ti,kw.

40 ((hazardous or toxic or toxicity or toxin or toxins or risk or risks or impact or impacts) adj5 environment*).ti,ab,kw.

41 (natural environment* or soil or soils or flora or floras or fauna or faunas or renewable resource*).ti,kw.

42 (waste* or contamination*).ti,kf.

43 (pollution* or polluting or pollutant* or contaminated orenvironmental health).ti,ab,kf.

44 (natural environment* or soil or soils or flora or floras or fauna or faunas or renewable resource*).ti,ab,kf.

45 (environment* adj5 sustainabilit*).ti,ab,kf.

46 carbon footprint*.ti,ab,kf.

47 (environment* adj2 (assess* or impact* or outcome* or implication* or consideration*)).ti,ab,kf.

48 environment*.ti,kf.

49 environmental*.ab.

50 ((health technology assessment or HTA or HTAs) andenvironmental*).ti,ab,kf.

51 ((health technology assessment or HTA or HTAs) adj7environment).ti,ab,kf.

52 38 or 39 or 40 or 41 or 42 or 43 or 44 or 45 or 46 or 47 or48 or 49 or 50 or 51

53 29 and 30

54 31 or 32 or 33 or 34 or 37

55 29 and 54

56 29 and 52

57 limit 55 to yr="2011 -Current"

58 limit 55 to yr="2016 -Current"

60 limit 53 to yr="2016 -Current"

61 limit 56 to yr="2016 -Current"

EMBASE

#67 #61 AND [2016-2021]/py

#66 #34 AND #60 AND [2016-2021]/py

#65 #34 AND #60

#64 #34 AND #62 AND [2016-2021]/py

#63 #34 AND #62

#62 #36 OR #37 OR #38 OR #39 OR #40

#61 #34 AND #35

#60
#41 OR #42 OR #43 OR #44 OR #45 OR #46 OR #47 OR #48 OR #49 O
R #50 OR #51 OR #52 OR #53 OR #54 OR #55 OR #56 OR #57 OR #58
OR #59

#59
(('health technology assessment' OR hta OR htas) NEAR/7 environment):ti,ab,de

#58 ('health technology assessment':ti,ab,de OR hta:ti,ab,de OR htas:ti,ab,de) AND environmental*:ti,ab,de

#57 environmental*:ab

#56 environment*:ti,de

#55 (environment* NEAR/2 (assess* OR impact* OR outcome* OR implication* OR consideration*)):ti,ab,de

#54 'carbon footprint*':ti,ab,de

#53
(environment* NEAR/5 sustainabilit*):ti,ab,de

#52 pollution*:ti,ab,de OR polluting:ti,ab,de OR pollutant*:ti,ab,de OR contaminated:ti,ab,de OR 'environmental health':ti,ab,de

#51 waste*:ti,de OR contamination*:ti,de

#50 'natural environment*':ti,kw OR soil:ti,kw OR soils:ti,kw OR flora:ti,kw OR floras:ti,kw OR fauna:ti,kw OR faunas:ti,kwOR 'renewable resource*':ti,kw

#49
((hazardous OR toxic OR toxicity OR toxin OR toxins OR risk OR risks OR impact OR impacts) NEAR/5 environment*):ti,ab,kw

#48 waste*:ti,kw OR pollution*:ti,kw OR polluting:ti,kw OR pollutant*:ti,kw OR contamination*:ti,kw OR contaminated:ti,kwOR 'environmental health':ti,kw

#47 'environmental health'/exp
#46 'biota'/exp
#45 'ecotoxicology'/exp
#44 'environmental impact'/exp
#43 'environmental exposure'/exp
#42 'pollutant'/exp
#41 'pollution'/exp
#40 'patient attitude'/de OR 'patient participation'/de OR 'patient decision making'/de OR 'patient preference'/de OR 'patient satisfaction'/de OR 'patient centered' OR patientcentered OR 'profesional patient*' OR (((patient* OR individual* OR person* OR carer OR caregiver* OR client OR communit* OR consumer* OR public* OR user* OR sufferer* OR suff ering OR diseased OR troubled OR sick* OR people OR patient OR pati ents OR proband* OR individuals OR survivor* OR family OR families O R familial OR kindred* OR relative OR relatives OR 'care giver*' OR caregiver* OR carer OR carers OR personal OR spous* OR p artner OR partners OR couples OR users OR participant* OR people OR child* OR teenager* OR adolescent* OR youth OR girls OR boys OR adults OR elderly OR females OR males OR women* OR men OR mother
* OR father* OR parents OR parent OR parental OR maternal OR patern al) NEAR/2 ('service user' OR preference* OR opinion OR perception* OR perspective* OR vi ew* OR voice* OR deerience* OR detectation* OR wish OR wishes OR attitud* OR wellbeing OR 'well being' OR 'selfcare' OR self* OR belief* OR concern* OR worr* OR burden* OR proble m* OR distress OR psychology* OR 'social activit*' OR famil* OR friend* OR emotion* OR satisf* OR dissatisf* OR h app* OR unhapp* OR behav* OR lifestyle OR routine OR life OR autono my OR 'activities of daily living' OR 'quality of life' OR 'everyday life' OR skeptic* OR enthusias* OR 'daily lives' OR 'frequent or daily users' OR acceptanc*)):ab) OR (((patient* OR individual* OR person* OR carer OR caregiver* OR client OR communit* OR consumer* OR public* OR user* OR sufferer* OR suff ering OR diseased OR troubled OR sick* OR people OR patient OR pati ents OR proband* OR individuals OR survivor* OR family OR families O R familial OR kindred* OR relative OR relatives OR 'care giver*' OR caregiver* OR carer OR carers OR personal OR spous* OR p artner OR partners OR couples OR users OR participant* OR people OR child* OR teenager* OR adolescent* OR youth OR girls OR boys OR a

dults OR elderly OR females OR males OR women* OR men OR mother
* OR father* OR parents OR parent OR parental OR maternal OR patern
al)

NEAR/
5
(prescrib* OR elicit* OR choice* OR logit OR adheren* OR preferen* OR'service user' OR preference* OR opinion* OR perception* OR perspective* OR view* OR voice* OR experienc* OR expectation* OR wish OR wishes O R attitud* OR lifespan* OR refus* OR 'patient autonomy' OR 'activitiesof daily living' OR 'quality of life' OR 'everydaylife' OR decisi* OR paticipat* OR involvement* OR desir* OR activation OR empower* OR adaptat* OR educat*)):ti)

#39 'social acceptance'/mj OR 'social aspect'/mj OR 'social norm'/mj OR 'social problem'/mj OR 'social rejection'/mj OR 'social status'/mj OR 'social structure'/mj OR 'social aspects and related phenomena'/mjOR social:ti,de OR 'social factor':ti OR 'social aspect*':ti OR 'social norm':ti OR 'social burden*':ti OR 'social impact*':ti OR (((choice* OR 'information needs' OR communication* OR 'self care' OR 'self management' OR 'trade off' OR planning) NEAR/2 (social OR patient*)):ab)

#38 'organization'/mj OR 'personnel management'/mj OR 'professional practice'/mj OR 'organization and management'/mj OR 'healthcare common procedure coding system'/mj OR 'case manager'/mj OR 'program development'/mj OR organizational:ti,de OR (((organization* OR workflow OR 'work flow' OR 'workplanning' OR implementation* OR 'information needs' OR 'need assessment*' OR skills OR attitud* OR culture OR 'patient path' OR 'user path' OR 'client path' OR 'qualityassurance' OR sustainability OR centralization OR decentralization OR ' hospital managesocialment or manager*' OR supplier) NEAR/3 (patient* OR client* OR user OR users OR practice* OR hospital* OR home* OR 'primary care' OR clinical OR medical OR nurse* OR physician* OR profesional*)) :ab)

#37 'privacy'/exp OR 'fraud'/mj OR 'government regulation'/expOR 'health care disparity'/mj OR 'health care delivery'/mj OR 'humanism'/mj OR 'human rights'/mj OR 'insurance'/mjOR 'reimbursement'/mj OR 'jurisprudence'/exp OR 'law'/exp OR 'legislation and jurisprudence'/exp OR 'patient right'/exp OR 'prejudice'/mj OR 'professional autonomy'/mj OR 'professional misconduct'/mj OR (((healthcare OR 'health

care' OR nonclinical OR 'community based')
NEAR/1(deliver* OR distribution*)):ti,de) OR
liabilit*:ti,de OR litigation*:ti,deOR constitutional:ti,de
OR laws:ti,de OR jurisprudence:ti,de
OR complicit*:ti,de OR private:ti,de OR confidential*:ti,de OR
(((care OR treatment) NEAR/2 (duty OR obligat*)):ti,de)
OR((social* NEAR/1 (responsibl* OR
obligat*)):ti,de) OR(((informed OR presumed) NEAR/2 (choice
OR 'decision making')):ti,de)OR ((('basic right*' OR 'access* right' OR
'duty to know' OR 'equally access*' OR 'external
pressure' OR 'fundamental right*' OR 'human
right*' OR 'informed choice' OR 'informed decision making' OR
'public pressure' OR 'regulatory frame*' OR 'right of access*' OR
'right to know' OR acceptance OR accessibility OR accountability OR
autonomyOR beneficence OR 'civil right*' OR
communitarian* OR 'community values' OR confidentiality OR dignity
OR directive OR disclosure OR discrimination OR elsi OR elsis OR
equality OR equity OR fairness OR free dom OR harm OR inequalit*
OR inequity OR 'patient*
integrity' OR justice OR law OR lawmaker* OR lawsuit* OR lawyer* OR
legacy OR legal* OR legislation OR legitimacy OR liability OR litigation
O R medicolegal OR 'non coverage' OR nonmaleficence OR 'non
maleficence' OR normative OR normativity OR permissibility OR prejudi
ce* OR privacy OR reimburse* OR 'social
values' OR stigma OR stigmas OR stigmatisation OR stigmatise OR
stigmatization OR stigmatize OR transparency OR uncertainty OR
'value judgment') NEAR/5 ('basic right*' OR 'access* right' OR
'duty to know' OR 'equally access*' OR 'external
pressure' OR 'fundamental right*' OR 'human
right*' OR 'informed choice' OR 'informed decision making' OR
'public pressure' OR 'regulatory frame*' OR 'right of
access*' OR 'right to
know' OR acceptance OR accessibility OR accountability OR
autonomyOR beneficence OR 'civil right*' OR communitarian* OR
'community values' OR confidentiality OR dignity OR directive OR
disclosure OR discrimination OR elsi OR elsis OR equality OR equity
OR fairness OR free dom OR harm OR inequalit* OR inequity OR
'patient*
integrity' OR justice OR law OR lawmaker* OR lawsuit* OR lawyer* OR
legacy OR legal* OR legislation OR legitimacy OR liability OR litigation
O R medicolegal OR 'non coverage' OR nonmaleficence OR 'non
maleficence' OR normative OR normativity OR permissibility OR prejudi
ce* OR privacy OR reimburse* OR 'social
values' OR stigma OR stigmas OR stigmatisation OR stigmatise OR stig

matization OR stigmatize OR transparency OR uncertainty OR 'value judgment')):ti,de)

#36 'ethics'/exp OR ethic*:ti,de OR bioethic*:ti,de OR 'unintended consequences':ti,de OR 'skills and training':ti,de OR 'benefits and harms':ti,de OR beliefs:ti,de OR 'patient* autonomy':ti,de OR dignity:ti,de OR moral*:ti,de OR religio*:ti,de OR 'human rights':ti,deOR humanism:ti,de OR 'patient* integrity':ti,de OR principlism:ti,de OR normativ*:ti,de OR 'principle base*':ti,de OR beneficence:ti,de OR 'non maleficence':ti,de OR philosoph*:ti,de OR aristoteles:ti,de OR socrates:ti,de OR justice:ti,de OR fairness:ti,de OR 'patient* expectation*':ti,de OR 'patient* accessibility':ti,de OR beauchamp:ti,de OR childress:ti,de OR 'wide reflective*':ti,de OR socratic:ti,de OR 'social shaping':ti,de OR casuistry:ti,de OR 'coherence analy*':ti,de OR eclectic*:ti,de OR 'right to die':ti,de OR 'right to life':ti,de OR 'social value*':ti,de OR 'ethnic value*':ti,de OR 'personal value*':ti,de OR 'benefit harm':ti,de OR 'harm benefit':ti,de OR elsi:ti,de OR elsa:ti,de#35

((('semi structured' OR semistructured OR unstructured OR informal OR 'in-depth' OR indepth OR 'face-to-face' OR structured OR guide) NEAR/3 (interview* OR discussion* OR questionnaire*)):ti,de)OR (focus:ti,deAND group*:ti,de) OR qualitative:ti,de OR ethnograph*:ti,de OR fieldwork:ti,de OR 'field work':ti,de OR 'key informant':ti,de OR 'qualitative research'/exp

#34 #29 OR #33

#33 #30 OR #31 OR #32

#32 ((telemedicine OR telehealth*) NEAR/3 neurolog*):ti,ab#31 'teleneurology'/exp OR 'teleneurology'

#30 'telestroke'/exp OR 'telestroke'

#29 #13 AND #28

#28

#14 OR #15 OR #16 OR #17 OR #18 OR #19 OR #20 OR #21 OR #22 O R #23 OR #24 OR #25 OR #26 OR #27

#27 'motor dysfunction'/mj

#26 'multiple sclerosis'/mj

#25 'parkinson disease'/mj

#24 'epilepsy'/mj

#23 'cerebrovascular accident'/mj

#22 'movement disorder*':ti,ab

#21 ('nervous system' NEAR/1 (disorder* OR disease*)):ti,ab

#20 'multiple sclerosis*':ti,ab

#19 parkinson*:ti,ab

#18 epileps*:ti,ab

#17 stroke:ti,ab

#16 (neurological NEAR/1 (disorder* OR disease*)):ti,ab#15 'neurologic disease'/mj

#14 'neurology'/exp

#13

#1 OR #2 OR #3 OR #4 OR #5 OR #6 OR #7 OR #8 OR #9 OR #10 OR #11 OR #12

#12 ((mobile OR cell$ OR smart$) NEAR/1 (app$ OR device OR phone)):ti,ab

#11 'mobile application'/mj

#10 videoconferencing:ti,ab OR conferencing:ti,ab OR videoconference$:ti,ab OR 'video-conferenc$':ti,ab OR videoconsultation:ti,ab OR 'video-consultation':ti,ab

#9 'videoconferencing'/exp

#8 mhealth:ti,ab OR ehealth:ti,ab OR 'e-health':ti,ab OR 'm-health':ti,abOR 'm health':ti,ab OR 'mobile health':ti,ab

#7 teleconsult*:ti,ab OR telecare:ti,ab OR telemed*:ti,abOR telerehab*:ti,ab OR telehealth*:ti,ab OR 'tele-medicine':ti,ab OR 'tele medicine':ti,ab OR telehealth:ti,ab OR 'tele-health':ti,ab OR telehomecare:ti,ab OR 'tele-homecare':ti,ab

#6 'e-mail'/mj

#5 'telephone'/mj

#4 'remote sensing'/mj

#3 'computer network'/mj

#2 'teleconsultation'/mj

#1 'telemedicine'/mj
