# Supplementary material for: Ethical, Legal, Organisational and Social Issues of Teleneurology: A Scoping Review
Source: Int J Environ Res Public Health. 2023 Feb 19;20(4):3694. doi: 10.3390/ijerph20043694 (PMC9962592; doi:10.3390/ijerph20043694)
Supplement: Supplementary file 1 [file ijerph-20-03694-s001.zip › Supplementary File S4.pdf]

Author, year, country	Objective	Methodological Design	Population	Main finding	Conflicts of interest
Andrews, 2020, United Kingdom	To understand the perspectives of the use of TN in patients with multiple sclerosis, depression and epilepsy	Mixed study	Multidisciplinary healthcare professionals of primary and secondary care	Acceptability, barriers, perspective of professionals	Yes
Arighi, 2021, Italia	To describe the digital divide of a population of patients with dementia contacted by telemedicine during Italian lockdown for COVID-19 pandemic.	Prospective observational study	Patients with cognitive impairment and caregivers	Barriers	No
Bagot, 2016, Australia	Identify the benefits of a telemedicine service for acute stroke from an organizational and social perspective	Qualitative study	Telemedicine managers for patients presenting with symptoms of acute stroke	Organizational aspects, implementation; acceptability; barriers	No
Bagot, 2017, Australia	To compare the experiences of Australian and UK specialists in providing remote telemedicine consultations for patients with acute stroke.	Qualitative study	Specialists in telestroke	Professional perspective	Yes
Bagot, 2017, Australia	Summarize the factors that facilitated the statewide transition of The Victorian Stroke Telemedicine (VST) program	Implementation report	Stroke patients	Organizational aspects	No
Bagot, 2020, Australia	To identify factors that predicted intentions to use an acute stroke telemedicine service, before and after implementation for nurses and non-nurses; and identify post-implementation strengths and any improvements in 16 hospitals.	Mixed study	Professionals involved in acute stroke care	Barriers and facilitators: perceptions of telemedicine and technology; cultural and organizational environment; clinical care process, perceptions	No
Bagot, 2020, Australia	Identify factors to support the sustainability of an innovative stroke TN program	Qualitative study (Case study)	Emergency and intensive care physicians, urgency physician's physicians nurse and neurologists	Facilitators in the organizational environment, clinical care processes and the benefits of their use; barriers	No
Banks, 2021, Ireland	To understand the perceptions of chronic epilepsy management via telephone consultations from a clinician and patient perspective	Mixed study	Clinicians and patients	Experience of clinicians and patients	No
Bower 2021, Australia	Investigate the factors that influence the use of technology by doctors working in neurorehabilitation	Qualitative study	Health professionals; physical therapists and occupational therapists	Acceptability of professionals; organizational factors	No

Bruno, 2018, United Kingdom	To assess the access of people with epilepsy to digital and wearable technology and to identify the factors that influence the acceptability of digital tools in epilepsy care. Also, explore the perspective of caregivers and health professionals	Descriptive study	People with epilepsy, caregivers and health professional	Acceptability of patients, caregivers and professionals	No
Calleja-Castillo, 2018, Mexico	Describe the different roles that WhatsApp has acquired as a clinical tool in stroke systems and potential privacy concerns with its use	Narrative review	Stroke patients	Legal aspects and organizational communication	No
Casares, 2020, EE. UU	To evaluated perceptions in patients with epilepsy and providers during the COVID-19 pandemic	Descriptive study	Patients with epilepsy and providers	Perceptions in patients with epilepsy and providers; accessibility; barriers	No
Caughlin 2020, Canada	Identify facilitators and barriers to the implementation of telerehabilitation services in a research conte	Qualitative study	Stroke patients	Acceptability; facilitators and barriers	Yes
Chan, 2021, Canada	To explore the experiences of older stroke patients with video home visits at follow-up and understand the associated determinants, barriers and benefits	Qualitative study	Stroke patients	Acceptability, facilitators and barriers	Yes
Chen, 2020, EE. UU	To investigate the benefits perceived by patients and the barriers to using the telerehabilitation system at home	Qualitative study	Stroke patients	Acceptability and patient perspective	No
Cheshire, 2020, EE. UU	To assess patients' perceptions of physician empathy during telemedicine consultations compared to face-to-face consultations during an emergency assessment of acute stroke.	Cohort study	Adult patients receiving emergency department (stroke) consultation.	Acceptability	Yes
Chirra, 2019, EE.UU	To review studies evaluating telemedicine as a means to facilitate access to care, highly specialized visits, diagnostic consultations, rehabilitation and remote follow-up of neurological disorders.	Narrative review	Neurology’s patient	Acceptability	No
Cilia, 2020, Italy	To describe un a two-step model based on the COVID-19 experience	Observational (Case/control)	Patients with Parkinson’s	Organizational communication, information	No
Courtney, 2021, United Kingdom	To provide a more nuanced account of the experiences of clinicians with teleneurolgy	Qualitative study	Clinicians	Professionals’ perspective	No

Espay, 2019, EE. UU	Propose a roadmap for the development of patient-centred digital outcomes and their integration into both clinical care and research that is sensitive to the needs of all stakeholders, primarily patients	Narrative review	Parkinson's patients	Implementation issues	No
Fisher, 2016, United Kingdom	To assess the acceptability of wrist sensors in a sample of Parkinson's patients after short-term and prolonged use	Mixed study	Parkinson's patients	Acceptability	No
Gibson, 2016, United Kingdom	To explore the views of patients and carers on their experiences of using a stroke telemedicine system	Qualitative study	Stroke patients and carers	Perspective patient and caregiver; acceptability	No
Giunti, 2018, España	Exploring the needs of people with multiple sclerosis for physical activation To detect perceived barriers and facilitators of mHealth solutions in people with multiple sclerosis and healthcare professionals. To understand the motivational aspects behind the adoption of mHealth solutions for multiple sclerosis.	Mixed study	Patients with multiple sclerosis and healthcare professionals	Acceptability; barriers	No
Kjelle, 2021, Noruega	To explore how radiologists, paramedics and young doctors experience telemedicine-based stroke assessment and treatment	Qualitative study	Radiologists, paramedics and junior doctors	Organizational, implementation aspects	No
Kumar, 2021, United Kingdom	To describe the experience of video consultation during the COVID-19 pandemic in elderly population for Parkinson´s	Quality improvement report	Patients with elderly Parkinson’s	Organizational aspects; experience of patients	No
Lawson, 2020, Australia	Exploring the acceptability of the telerehabilitation experience to healthcare providers and patients in a memory rehabilitation program	Qualitative study	Stroke patients and clinicians	Acceptability	No
Liddle, 2016, Australia	Exploring Australian ethical and legal perspectives on the potential benefits of smartphone use while preserving privacy	Narrative review	Parkinson's patients	Ethical and legal issues	No
Macchi, 2021, EE. UU	To generate a person-centered description of the impact of COVID-19 from the perspectives of patients living with neurodegenerative disease and caregivers to improve outpatient palliative care delivery	Qualitative study	Patients with Parkinson`s disease, Alzheimer´s disease or related disorders and caregivers	Patients and caregiver perspectives; barriers	No

Mammen, 2016, EE. UU	To determine how patients and physicians perceive virtual visits and to identify components contributing to positive and negative perceptions	Qualitative study	Parkinson's patients and Parkinson's clinicians	Practitioner and patient perceptions of virtual visits, difficulties with the technology	No
McKenna, 2020, Ireland	To ascertain the patient's perspective of the experience of Remote Neurology Consultations during the COVID-19 Pandemic	Descriptive study	Neurology's patient	Acceptability; Patients perspectives	No
Morse, 2020, United Kingdom	To explore user perspectives on the use of self-administered virtual reality and identify barriers and facilitators before extending its use remotely as a telerehabilitation tool	Mixed study	Stroke patient's caregivers and clinicians	Acceptability, barriers and facilitators; perspective of patients, caregivers and practitioners	Yes
Nemeth, 2016, EE. UU	To identify and analyze individual and interpersonal barriers, facilitators and implications of health and community systems for improving acute stroke care and develop a multi-component community model for improving acute stroke care	Descriptive study	Recent stroke survivors, family members, clinicians	Acceptability, barriers and facilitators	No
O'Neil, 2018, Germany, Italy and Spain	To provide information on the adoption of virtual reality by 3 European clinics in 3 different service delivery models	Project report	Patients with motor impairment, as a secondary outcome of chronic neurological disease	Acceptability; implementation; barriers	No
Patel, 2019, EE. UU	Examine the current types of facilities available in teleneurology, as well as the outcomes, barriers, limitations, legal aspects and multidisciplinary aspects	Narrative review	Neurological patients	Acceptability	No
Rametta, 2020, EE. UU	To assess the rapid implementation of child neurology telehealth outpatient care with the onset of the coronavirus disease 2019 (COVID-19) pandemic in March 2020	Retrospective observational study	Child neurology	acceptability; perspectives patients and caregivers	No
Remy, 2020, Belgium	To identify access to telecommunication technologies and rehabilitation services for patients with multiple sclerosis and their willingness to use these technologies for rehabilitation	Cross-sectional study	Patients with multiple sclerosis	Acetpability; barriers	No
Saliba-Gustafsson, 2020, EE. UU	To evaluate the accelerated implementation of video visits in ambulatory neurology during the COVID-19 pandemic	Mixed study	Clinicians	Acceptability; appropriateness and sustainability	No

Sennott, 2020, EE. UU	To describe the program and practical strategies providers can implement to support wellbeing and successful telehealth uptake during this time of social isolation and gradual reopening.	Descriptive study	Patients with advanced Parkinson's disease or related disorders and caregivers	Acceptability; Organizational aspects; barriers	No
Shea, 2019, EE.UU	Examine the decision-making process and strategies employed during development, implementation and sustainability in the telestroke network.	Qualitative study	Representatives of hospitals with a telestroke programme and others not participating in telestroke	Implementation aspects	No
Simblett, 2019, United Kingdom, Spain and Italy	To investigate possible barriers and facilitators to the acceptability and sustained use of m-health in people with relapsing remitting sclerosis and progressive multiple sclerosis in different countries.	Qualitative study (Focus group)	Patients with multiple sclerosis	Acceptability; barriers	No
Simblett, 2019. United Kingdom, Spain and Italy	To identify potential uses, barriers and enablers for engagement with mHealth technology in people diagnosed with epilepsy in three European countries (Italy, UK and Spain).	Qualitative study	Patients with epilepsy	Acceptability	No
Simblett, 2020, United Kingdom	Conduct a consultation on what might be important to measure in studies using remote sensing technology, based on the experiences of people with chronic diseases	Qualitative study	Patients with depression, multiple sclerosis and epilepsy	Identification of several factors important for health and well-being	No
Spindler, 2020, USA	To describe experience as a template for other departments and practices seeking to establish teleneurology programs, as well as an illustration of the challenges and barriers to its implementation.	Report	Neurological patients	Organizational aspects; barriers	No
Taddei, 2020, Italy	To describe the program and practical strategies providers can implement to support wellbeing and successful telehealth uptake during this time of social isolation and gradual reopening	Mixed study	Children with neurodevelopmental disorders	Barriers; acceptability; perspectives patients	No
Terio, 2019, Uganda	To evaluate the implementation process of a family-centered rehabilitation intervention supported by a cell phone and gain insights into the mechanisms of impact, as well as contextual factors that might have affected the implementation process and its outcome	Qualitative study	Professionals stroke. Occupational therapists, researchers, information technology (ICT) specialists and rehabilitation managers	Practitioner perception, acceptability, barriers to implementation	No
Uscher-Pines, 2020, EE. UU	Comparing the characteristics and practices of emergency departments with robust and low telestroke assimilation.	Qualitative study	Emergency department representatives.	Practitioner perspective, organizational aspects	No

Velásquez, 2016, EE. UU	To analyze how the epilepsy service needs of rural communities are met with videoconferencing-centered clinics at the University of Kansas Telemedicine and Telehealth Center. Exploring resource shortages in rural área	Narrative review	Children with epilepsy in rural areas	Organizational, ethical and legal aspects	No
Vloothuis, 2018, EE.UU	Describe the content of the intervention "CARE4STROKE program (C4S)" intervention in detail and explain the implementation of this intervention in practice using the template for the intervention description and replication checklist	Descriptive study	Stroke patients and caregivers	Organizational aspects	No
von Wrede, 2020, Germany	N/A	Mixed study	Patients with epilepsy	Barriers; acceptability; perspectives patients	Yes
Wannheden, 2020, Sweden	To explore the expectations of patients and health professionals about eHealth functionalities for co-care.	Qualitative study	People with Parkinson's disease and healthcare professionals	Expectations of patients and professionals, organizational constraints, barriers	No
Wentink, 2019, Holland	Identify user requirements for a comprehensive eHealth program in stroke rehabilitation.	Qualitative study	Stroke patients, caregivers, health care professionals	Users' and professionals on perspective; accessibility	No
Whetten, 2019, Mexico	Analyze the impact of travel and emissions avoided as a result of neuro-emergency telemedicine consultations.	Report	N/A	Greenhouse gas reduction using teleneurology consultations	No
Yang, 2020, EE. UU	To inform strategies to sustain neurology’s expanded use of this modality and inform standards or guidelines for patients who are clinically appropriate for video visits.	Report	Neurology Outpatients	Organizational aspects; barriers; acceptability	No
